# Conceptual Rationale for Combining Galantamine, Iontophoresis, and Black Sea Brine in Peripheral Neuropathy: A Narrative Review

**DOI:** 10.3390/neurosci7030060

**Published:** 2026-05-19

**Authors:** Mariya Ivanova, Liliya Panayotova-Ovcharova, Detelina Nedyalkova-Petkova, Petar Petkov, Georgi Boshev, Evgeniya Vladeva

**Affiliations:** 1 Faculty of Pharmacy, Medical University of Varna, 9000 Varna, Bulgaria; 2 Department of Physiotherapy, Rehabilitation and Thalassotherapy, Medical University of Varna, 9000 Varna, Bulgaria; l.panayotova@mu-varna.bg (L.P.-O.); detelina.petkova@mu-varna.bg (D.N.-P.); jeni.vladeva@gmail.com (E.V.); 3 Department of Orthopaedics and Traumatology, Medical University of Varna, 9000 Varna, Bulgaria; drpeturpetkov@gmail.com; 4 Department of Neurosurgery and ENT, Medical University of Varna, 9000 Varna, Bulgaria; dr.g.boshev@gmail.com

**Keywords:** galantamine, iontophoresis, Black Sea brine, peripheral neuropathy, transdermal drug delivery, rehabilitation, narrative review

## Abstract

Background: Peripheral neuropathy is a common and clinically heterogeneous neurological condition caused by metabolic, inflammatory, toxic, or traumatic factors and is associated with sensory deficits, neuropathic pain, motor impairment, and reduced functional capacity. Management remains challenging and often requires multimodal therapeutic approaches, as pharmacological monotherapy frequently provides incomplete symptom control. Objective: This narrative review explores the conceptual rationale for combining galantamine with iontophoresis and Black Sea brine-based therapy as a potential multimodal strategy for peripheral neuropathy management. Main Findings: Galantamine, a reversible acetylcholinesterase inhibitor and positive allosteric modulator of nicotinic acetylcholine receptors, has demonstrated neuroprotective, neuromodulatory, and anti-inflammatory properties in experimental settings. Iontophoresis may provide a non-invasive method for targeted local drug delivery while reducing systemic exposure. Black Sea brine, widely used in Bulgarian balneological and rehabilitation practice, has been associated with improved circulation, pain reduction, and neuromuscular support. The reviewed evidence suggests biologically plausible complementary mechanisms; however, no direct clinical studies evaluating the combined intervention were identified. Limitations: Current evidence is indirect and derived from separate investigations of galantamine, iontophoresis, and brine-based therapy, as well as heterogeneous historical and regional sources. Therefore, the proposed combination should be considered hypothesis-generating rather than evidence-established. Conclusions: The combination of galantamine, iontophoresis, and Black Sea brine represents a potentially interesting multimodal concept for peripheral neuropathy rehabilitation. Well-designed preclinical and clinical studies are required to determine safety, feasibility, optimal treatment parameters, and therapeutic efficacy.

## 1. Introduction

Peripheral neuropathy is a prevalent and clinically significant condition encountered in neurology, internal medicine, and physical medicine and rehabilitation [1]. It arises from a wide range of etiologies, including metabolic disorders, inflammation, toxic exposure, nutritional deficiencies, chemotherapy, and traumatic nerve injuries [2,3]. Patients commonly present with sensory disturbances, neuropathic pain, motor impairments, autonomic dysfunction, and functional limitations, all of which may substantially reduce mobility, independence, and overall quality of life [4,5]. In addition, recent reviews emphasize the growing global burden of neuropathy, particularly in aging populations and individuals with chronic metabolic diseases [6].

Peripheral neuropathy should not be considered a single clinical entity but rather a heterogeneous syndrome comprising multiple subtypes with distinct etiologies and pathophysiological mechanisms. Common forms include diabetic peripheral neuropathy, compressive or traumatic neuropathies, inflammatory neuropathies, toxic neuropathies, and chemotherapy-induced peripheral neuropathy [2,3]. This heterogeneity contributes to variability in disease progression, symptom profiles, and response to treatment, complicating clinical management.

Current therapeutic strategies focus primarily on addressing the underlying cause, symptomatic pain control, and functional rehabilitation. Frequently used pharmacological agents include anticonvulsants such as pregabalin and gabapentin, antidepressants such as duloxetine and tricyclic antidepressants, topical agents, and analgesics [3]. However, these treatments often provide only partial symptom relief and are frequently associated with adverse effects, resulting in suboptimal adherence and persistent disability [7]. Importantly, most available therapies do not directly target the underlying mechanisms of nerve injury or promote nerve regeneration.

Pharmacological treatment remains a cornerstone of peripheral neuropathy management. Galantamine, a reversible acetylcholinesterase inhibitor and positive allosteric modulator of nicotinic acetylcholine receptors, is primarily used in cognitive disorders such as Alzheimer’s disease [8]. Beyond its central effects, emerging experimental evidence suggests that galantamine may exert neuroprotective, anti-inflammatory, and neuromodulatory actions in the peripheral nervous system [9,10]. These effects are thought to be mediated, at least in part, through activation of the cholinergic anti-inflammatory pathway and modulation of neuroinflammatory signaling. Nevertheless, clinical evidence supporting its use in peripheral neuropathy remains limited, and its application in this context should currently be considered exploratory [9].

In parallel with pharmacological approaches, physical medicine and rehabilitation interventions play an essential role in the management of peripheral neuropathy [3]. Brine-based therapies, including Black Sea brine traditionally used in Bulgarian rehabilitation practice, have been applied in musculoskeletal, neurological, respiratory, and chronic pain conditions, with reported benefits in circulation, pain reduction, functional recovery, and quality of life [11]. These effects are often attributed to the mineral composition of brine, including magnesium and other bioactive components, as well as thermal and osmotic influences. However, despite long-standing clinical use and encouraging regional observations, high-quality controlled evidence supporting its role in peripheral neuropathy remains limited.

Iontophoresis is a non-invasive technique that uses low-intensity electrical current to facilitate the transdermal transport of ionized molecules [12,13,14]. It has been investigated as a method for localized delivery of therapeutic agents, offering potential advantages such as reduced systemic exposure, avoidance of injections, and improved targeting [14]. Recent technological developments in transdermal drug delivery systems, including digital control and advanced delivery platforms, further support its potential application in neurological conditions [15,16,17]. However, clinical evidence supporting iontophoresis in peripheral neuropathy remains limited and heterogeneous, and current applications are largely experimental or indirect [18].

In this context, iontophoresis may represent a translational strategy for targeted delivery of galantamine in selected peripheral neuropathic conditions. Recent narrative work highlights interest in non-systemic delivery approaches, including transdermal and iontophoretic administration of galantamine, but also emphasizes the absence of rigorous clinical trials [9].

The rationale for combining galantamine, iontophoresis, and Black Sea brine lies in their potentially complementary mechanisms of action, including cholinergic modulation, localized drug delivery, mineral-supported rehabilitation effects, and possible improvement of the local tissue microenvironment. Whether these interactions are additive, synergistic, or clinically meaningful remains unknown and requires systematic investigation.

Therefore, the aim of this narrative review is to critically examine the conceptual basis, available evidence, and translational feasibility of combining galantamine, iontophoresis, and Black Sea brine in peripheral neuropathy. Particular emphasis is placed on current limitations of evidence, safety considerations, and future research priorities.

## 2. Scope and Literature Sources

This article is presented as a narrative review. Relevant literature related to galantamine, iontophoresis, peripheral neuropathy, and Black Sea brine therapy was identified through targeted searches in major scientific databases, including PubMed, Scopus, and Google Scholar, as well as selected regional and historical sources.

Given the absence of direct clinical studies evaluating the combined application of galantamine, iontophoresis, and Black Sea brine, this review integrates indirect evidence derived from experimental research, clinical observations, and established rehabilitation practices. The aim is to synthesize current knowledge, identify translational gaps, and outline directions for future research.

## 3. Peripheral Neuropathy: Current Therapeutic Challenges

Peripheral neuropathy represents a heterogeneous group of disorders affecting the peripheral nervous system, with more than 200 identified etiologies and diverse clinical presentations [2,19,20]. It is increasingly recognized as a major global health burden, particularly in aging populations and individuals with chronic metabolic diseases, including diabetes mellitus, where prevalence continues to rise worldwide [21]. In addition to its high prevalence, peripheral neuropathy is associated with substantial healthcare costs, increased risk of disability, and reduced quality of life, highlighting its clinical and socioeconomic importance [22].

From a clinical and anatomical perspective, peripheral neuropathies are commonly classified according to the distribution and number of affected nerves into mononeuropathies, multifocal neuropathies (mononeuritismultiplex), and polyneuropathies, with distal symmetric polyneuropathy being the most prevalent form [2,23]. Additional classification systems consider the predominant fiber involvement (sensory, motor, autonomic) and underlying pathological mechanisms, including axonal degeneration, demyelination, or mixed patterns [3,20,24]. This multidimensional classification reflects the complexity of peripheral nerve disorders and has important implications for diagnosis, prognosis, and treatment selection.

Etiologically, peripheral neuropathy encompasses a wide spectrum of conditions. Diabetic peripheral neuropathy is the most common form globally and is strongly associated with metabolic dysregulation, oxidative stress, mitochondrial dysfunction, and microvascular impairment [21,24]. Traumatic and compressive neuropathies, such as entrapment syndromes, arise from mechanical injury or ischemia affecting individual nerves. Inflammatory and immune-mediated neuropathies, including Guillain–Barré syndrome and vasculitic neuropathies, involve dysregulated immune responses that lead to nerve damage. Toxic, infectious, hereditary, and chemotherapy-induced neuropathies further contribute to disease heterogeneity and clinical variability [4,19]. These diverse pathogenic mechanisms underscore the need for individualized and mechanism-based therapeutic approaches.

Clinically, peripheral neuropathy is characterized by a combination of neuropathic pain, sensory deficits, and motor dysfunction. Patients frequently report burning pain, paresthesia, allodynia, numbness, and progressive weakness, typically in a length-dependent “stocking–glove” distribution [5,7]. Neuropathic pain, in particular, represents a major therapeutic challenge due to its complex pathophysiology involving peripheral and central sensitization mechanisms [22,25,26]. In many patients, autonomic dysfunction may also be present, further complicating clinical presentation and contributing to increased morbidity.

Despite advances in understanding pathophysiological mechanisms, therapeutic options remain limited. Current management is largely symptomatic and includes anticonvulsants (e.g., pregabalin, gabapentin), antidepressants (e.g., duloxetine, tricyclic antidepressants), and topical agents. However, these treatments frequently provide only partial relief and are often associated with adverse effects, leading to suboptimal adherence and persistent disability [3,7,25,27]. Importantly, conventional therapies do not adequately address underlying disease mechanisms such as neuroinflammation, oxidative stress, or impaired nerve regeneration.

In recent years, increasing attention has been directed toward the mechanisms of peripheral nerve injury and regeneration. However, spontaneous regeneration is often incomplete and slow, particularly in chronic metabolic or inflammatory conditions [28]. This further contributes to persistent symptoms and functional impairment in affected patients.

These limitations highlight the need for novel, mechanism-based, and multimodal therapeutic strategies, particularly those targeting inflammation, neuroprotection, and tissue regeneration. In this context, the integration of pharmacological and rehabilitation-based approaches may represent a promising direction for future research [9,21]. Emerging concepts emphasize the importance of combining systemic and localized interventions to optimize therapeutic outcomes, improve nerve recovery, and enhance patient quality of life.

## 4. Galantamine: Pharmacological and Translational Rationale

Galantamine is a reversible acetylcholinesterase inhibitor and a positive allosteric modulator of nicotinic acetylcholine receptors, widely used in the management of cognitive disorders such as Alzheimer’s disease [8,16,29,30]. Its dual mechanism of action enhances cholinergic neurotransmission both by inhibiting acetylcholine degradation and by potentiating nicotinic receptor activity, thereby influencing synaptic transmission, neuronal excitability, and network-level signaling [31]. Beyond cognition, these properties suggest broader neuromodulatory potential, including effects on neuroinflammation and neuronal survival.

Increasing experimental and translational evidence indicates that galantamine may exert neuroprotective and neuromodulatory effects in the peripheral nervous system [9]. Proposed mechanisms include the enhancement of cholinergic signaling, attenuation of oxidative stress, modulation of inflammatory pathways, and support of neuronal plasticity and survival [10,31]. In particular, activation of the cholinergic anti-inflammatory pathway—mediated via vagal efferent signaling—has been implicated in the regulation of systemic and local inflammation, representing a key neuroimmune interface relevant to peripheral nerve injury and repair [10].

Furthermore, emerging evidence suggests that cholinergic modulation may influence peripheral nerve regeneration processes. Experimental studies indicate that cholinergic signaling can affect axonal sprouting, Schwann cell function, and synaptic reorganization, all of which are critical for nerve repair [28,32]. These findings support the biological plausibility of galantamine as a potential adjunctive agent in peripheral nerve regeneration, although direct clinical validation remains lacking.

Despite this mechanistic rationale, clinical evidence supporting the use of galantamine in peripheral neuropathy remains limited. Most available data derive from experimental studies, pharmacological analyses, or indirect clinical observations, and no large-scale randomized controlled trials have specifically evaluated its efficacy in this context [9]. Consequently, its application in peripheral neuropathy should currently be regarded as exploratory and hypothesis-generating rather than evidence-based.

In some regional clinical settings, galantamine is incorporated into therapeutic protocols for peripheral nerve disorders. According to national clinical practice recommendations, early treatment of neuritis and neuropathies is encouraged, ideally within the first 10–15 days (National Health Insurance Fund, 2024; available online: nhif.bg (accessed on 14 May 2026)). Treatment regimens may include intramuscular galantamine in combination with B-group vitamins and physiotherapy interventions (Koleva, 2011) [33]. In patients presenting with both sensory and motor deficits, cholinergic agents may be administered following structured ascending–descending dosing schedules over periods of 30–90 days. However, these approaches reflect regional practice patterns and are not supported by controlled clinical trials.

Real-world prescribing data further illustrate its clinical utilization. National Health Insurance Fund data indicate that galantamine is among the most frequently prescribed medications for diabetic polyneuropathy and related neurological conditions (National Health Insurance Fund, 2024). While such data provide insight into clinical practice, they do not constitute evidence of therapeutic efficacy or comparative effectiveness.

From a translational perspective, galantamine presents both opportunities and challenges for localized drug delivery. The molecule is positively charged, relatively small, and water-soluble, which theoretically favors transdermal transport. However, its physicochemical properties—including pH-dependent stability, ionization characteristics, and potential degradation—may complicate dosing precision and reproducibility [14]. In addition, electrotransport mechanisms such as electrorepulsion and electroosmosis play a critical role in iontophoretic drug delivery and are highly dependent on molecular properties and skin physiology [34,35].

Iontophoretic delivery of galantamine generally follows established electrotherapy protocols. An aqueous solution of galantamine hydrobromide is prepared at concentrations between 0.25% and 1.0%, with pH adjusted to slightly acidic or neutral values (approximately pH 5–6). Due to its positive charge, the drug is delivered under the anode, while the return electrode is positioned at a distant site to complete the circuit. A continuous galvanic current is applied, typically in the range of 0.5–5 mA depending on patient tolerance, with treatment sessions lasting 10–20 min and repeated over 10–15 procedures [12,13,36].

Despite these standardized approaches, clinical evidence supporting iontophoretic delivery of galantamine in peripheral neuropathy remains extremely limited. Available data are largely conceptual or derived from indirect applications in pain management and rehabilitation. Even for other iontophoretically delivered agents, such as ketamine or local anesthetics, clinical outcomes have been inconsistent and not disease-specific [18,37,38].

Recent advances in transdermal drug delivery systems—including controlled-release platforms, microneedle-assisted delivery, and digitally controlled iontophoretic systems—highlight the potential of non-systemic approaches for neurological disorders [15,16,17]. These technologies may improve drug targeting, dosing precision, and patient adherence, although their clinical applicability in peripheral neuropathy remains to be established.

Taken together, the pharmacological profile of galantamine—characterized by cholinergic enhancement, anti-inflammatory modulation, and potential neuroprotective effects—provides a strong conceptual foundation for its investigation in peripheral neuropathy. However, the absence of robust clinical evidence, variability in delivery methods, and lack of standardized protocols underscore the need for well-designed experimental and clinical studies to establish its safety, efficacy, and translational relevance.

## 5. Black Sea Brine: Biological and Rehabilitation Rationale

Black Sea brine is a highly mineralized natural product derived from coastal salt lake systems in Bulgaria, particularly in the Pomorie and Burgas regions. It is characterized by a complex ionic composition, including magnesium, chloride, sulfate, calcium, potassium, and trace elements with potential biological activity [39,40]. The physicochemical properties of brine—such as high salinity, osmotic pressure, and mineral saturation—are considered central to its therapeutic effects in balneology and rehabilitation medicine.

Within Bulgarian physical medicine and rehabilitation practice, Black Sea brine has long been applied through baths, local applications, compresses, and electrophysiological procedures [12]. These interventions are commonly integrated into comprehensive rehabilitation programs, particularly in patients with musculoskeletal and neurological conditions, including peripheral neuropathies [22,41]. Clinical observations suggest that brine-based therapies may contribute to pain reduction, functional recovery, and improved quality of life, although the level of evidence remains variable.

Several biological mechanisms have been proposed to explain the therapeutic effects of brine. These include local vasodilation and improved microcirculation, thermal and osmotic effects facilitating tissue exchange, reduction in muscle spasms, and modulation of inflammatory processes [39,42]. In addition, mineral components may influence skin receptors and peripheral nerve endings, potentially contributing to neuromodulation and analgesic effects [4,5].

**Figure 1:**
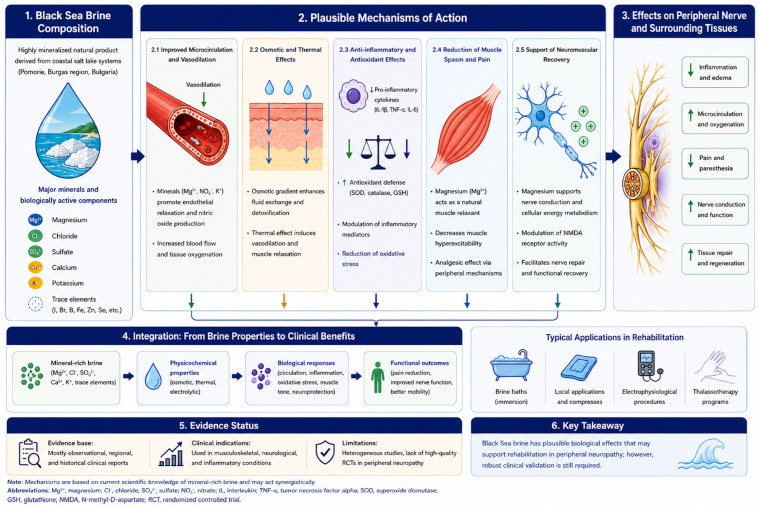
Mechanisms of action of Black Sea brine in peripheral neuropathy.

These interacting mechanisms are summarized in Figure 1, which illustrates the proposed pathways through which Black Sea brine may influence peripheral nerve function, including microcirculatory effects, anti-inflammatory modulation, and neuromuscular stabilization.

Figure 1 shows a schematic representation of the proposed biological and physiological mechanisms through which Black Sea brine may influence peripheral nerve function. The mineral-rich composition (e.g., magnesium, chloride, sulfate, calcium, potassium) may exert combined physicochemical and neurobiological effects, including improved microcirculation, modulation of inflammatory processes, reduction in muscle spasms, and support of neuromuscular recovery. Magnesium is highlighted as a key component due to its role in nerve conduction, NMDA receptor modulation, and potential contribution to neuroprotection and peripheral nerve regeneration. The illustrated pathways are based on experimental data, clinical observations, and mechanistic hypotheses, and should be interpreted as conceptual rather than definitively established.

Magnesium, one of the predominant ions in Black Sea brine, is of particular relevance due to its role in nerve conduction, neuromuscular excitability, and cellular energy metabolism. Experimental and translational studies indicate that magnesium may modulate NMDA receptor activity, reduce neuronal hyperexcitability, and exert anti-inflammatory effects [21,32]. Emerging evidence also suggests a potential role of magnesium in supporting peripheral nerve regeneration and repair processes, further strengthening the biological plausibility of brine-based interventions [32].

Regional clinical observations provide additional, although limited, support for the therapeutic relevance of Black Sea brine. A study conducted in Pomorie, Bulgaria, reported improvements in pain intensity and functional status in patients with diabetic polyneuropathy undergoing thalassotherapy programs that included brine applications [43]. Similarly, broader balneotherapy research has demonstrated beneficial effects in osteoarthritis, spinal disorders, and chronic inflammatory conditions, including improvements in circulation, pain, and functional outcomes [11,42].

Recent studies in balneology further emphasize the importance of mineral composition and environmental factors in therapeutic outcomes. Investigations into saline ecosystems and therapeutic lagoons suggest that combined climatic, chemical, and physical factors may exert synergistic effects on recovery processes [39]. Additional data from rehabilitation medicine indicate that integrated spa-based interventions may support systemic anti-inflammatory responses and improve patient-reported outcomes (Vitkovsky et al., 2025) [44].

Despite these promising findings, the current evidence base remains heterogeneous and limited. Most studies are observational, region-specific, or involve multimodal interventions, making it difficult to isolate the independent effect of brine. Variability in mineral composition, treatment protocols, and outcome measures further complicates comparisons across studies [42].

A structured overview of the available clinical and experimental evidence is presented in Table 1, which summarizes key studies investigating brine-based and balneotherapy interventions, their reported outcomes, and their methodological limitations.

The available evidence is heterogeneous and primarily derived from observational studies, regional clinical practice, and indirect rehabilitation settings.

Importantly, there is a lack of high-quality randomized controlled trials specifically evaluating Black Sea brine in peripheral neuropathy. Therefore, while Black Sea brine represents a biologically plausible and historically established component of rehabilitation practice, its role in peripheral neuropathy should be considered supportive and adjunctive rather than evidence-based.

Future research should focus on well-designed controlled trials, standardized treatment protocols, and mechanistic studies to clarify its therapeutic potential and optimize its integration into multimodal rehabilitation strategies [3,6].

## 6. Iontophoresis as a Local Delivery Strategy

Iontophoresis is a non-invasive transdermal drug delivery technique that uses low-intensity electrical current to facilitate the transport of ionized molecules across the skin barrier [12,13]. The underlying mechanisms include electrorepulsion (electromigration) and electroosmosis, which enable charged and, to a lesser extent, neutral molecules to penetrate the stratum corneum and reach deeper tissues [34,35]. These mechanisms distinguish iontophoresis from passive transdermal diffusion and allow controlled enhancement of drug delivery.

The technique offers several potential advantages, including localized drug administration, reduced systemic exposure, avoidance of injections, and the ability to modulate dosing through adjustable current parameters [35,36]. These features make iontophoresis particularly attractive in rehabilitation medicine and pain management, where targeted delivery to superficial tissues and peripheral nerves is desirable [3,37]. In addition, patient compliance may be improved compared to systemic pharmacotherapy, especially in chronic conditions.

However, the efficiency of iontophoretic delivery depends on multiple interacting factors. Drug-related properties such as molecular weight, charge, polarity, solubility, and stability play a critical role, as do physiological variables including skin hydration, regional blood flow, and interindividual variability in skin resistance [14,35]. Furthermore, technical parameters—such as current density, electrode configuration, treatment duration, and pH of the formulation—must be carefully controlled to ensure reproducibility and safety [34,36]. These complexities represent a major limitation in translating iontophoresis into standardized clinical protocols.

In the context of galantamine, several translational considerations are particularly relevant. Galantamine is a positively charged, relatively low-molecular-weight compound, which theoretically favors iontophoretic delivery under the anode [29,45]. However, its physicochemical characteristics, including pH-dependent stability and sensitivity to formulation conditions, complicate precise dosing and consistent tissue penetration [14]. In addition, variability in skin permeability and electrode–skin interface conditions may result in significant differences in delivered dose, raising concerns regarding reproducibility and therapeutic predictability.

Recent advances in transdermal drug delivery systems (TDDSs) have further expanded the potential of iontophoresis. Modern approaches include integration with microneedles, nanocarriers, and digitally controlled delivery platforms, which aim to improve drug permeation, spatial targeting, and dosing precision [16,17,46]. Hybrid systems combining iontophoresis with other technologies may overcome some of the limitations of conventional methods. Nevertheless, these innovations remain largely experimental in the context of neurological disorders and have not yet been validated in peripheral neuropathy [15].

Clinical evidence supporting iontophoresis in neuropathic conditions remains limited and heterogeneous. Studies investigating iontophoretic delivery of local anesthetics have demonstrated reversible cutaneous anesthesia in experimental settings, supporting the feasibility of targeted neural modulation [37]. Similarly, iontophoretic administration of S(+)-ketamine has been evaluated in a placebo-controlled trial for central neuropathic pain, showing limited analgesic benefit but acceptable tolerability [18]. In rehabilitation practice, iontophoresis has been used as an adjunct modality in conditions such as carpal tunnel syndrome, although clinical outcomes are inconsistent and often methodologically limited [38].

A structured overview of the available clinical and experimental evidence is presented in Table 2, which summarizes key studies involving iontophoresis-based interventions in neuropathic and related conditions, including their reported outcomes and limitations.

Overall, current data are derived primarily from experimental models, small clinical studies, or indirect applications in pain management and rehabilitation. Importantly, no robust clinical trials have specifically evaluated iontophoresis for galantamine delivery in peripheral neuropathy, and existing evidence remains conceptual or exploratory [6].

These limitations are consistent across different iontophoretically delivered agents. For example, both local anesthetics and ketamine demonstrate variable clinical effects depending on dosing protocols and patient-specific factors, highlighting the challenges in achieving consistent therapeutic outcomes [18,37].

Therefore, while iontophoresis represents a biologically plausible and technologically advanced method for localized drug delivery, its clinical application in peripheral neuropathy should currently be considered investigational. Future research should focus on optimizing drug formulations, standardizing delivery parameters, and conducting well-designed clinical trials to evaluate pharmacokinetics, safety, and therapeutic efficacy in neuropathic populations [16,35].

## 7. Proposed Combined Therapeutic Model

A conceptual multimodal therapeutic model can be proposed integrating pharmacological, physical, and rehabilitation-based interventions to target different aspects of peripheral nerve dysfunction. Within this framework, galantamine provides cholinergic modulation and potential anti-inflammatory and neuroprotective effects [10,29], iontophoresis enables localized and controlled transdermal drug delivery [14,34,35], and Black Sea brine may optimize the tissue microenvironment through mineral, thermal, and circulatory mechanisms [33,39].

The rationale for combining these modalities lies in their potentially complementary and multi-level mechanisms of action. Peripheral neuropathy is characterized by complex pathophysiological processes, including neuroinflammation, oxidative stress, impaired microcirculation, and defective nerve regeneration [2,7,21]. Galantamine may influence neurochemical signaling and inflammatory pathways through cholinergic modulation, including activation of the cholinergic anti-inflammatory pathway, which has been implicated in the regulation of systemic and local inflammation [10]. At the same time, iontophoresis offers a strategy to bypass systemic pharmacokinetics and deliver active compounds directly to the affected region, potentially enhancing local bioavailability while reducing systemic adverse effects [46].

In parallel, brine-based therapy may contribute to improving local tissue conditions by enhancing microcirculation, modulating inflammatory processes, and supporting neuromuscular recovery through mineral-mediated and thermal effects [39,44]. Magnesium-rich environments, in particular, have been associated with modulation of neuronal excitability and support of nerve regeneration processes, providing an additional mechanistic link between balneotherapy and neurorehabilitation [32].

From a systems perspective, this multimodal approach may simultaneously target biochemical, electrophysiological, and microenvironmental factors contributing to peripheral nerve dysfunction. Such an integrated strategy aligns with contemporary trends in neuropathic pain and neurorehabilitation research, which emphasize multimodal and mechanism-based interventions rather than single-agent therapies [6,25].

However, it is important to emphasize that the interaction between these modalities remains hypothetical. At present, no experimental or clinical studies have directly evaluated the combined use of galantamine, iontophoresis, and brine therapy. The potential interactions—whether additive, synergistic, or independent—are not yet understood, and the relationship between cholinergic modulation, transdermal transport dynamics, and mineral-mediated tissue effects has not been mechanistically validated [16].

Furthermore, variability in treatment protocols, patient characteristics, and disease subtypes may significantly influence therapeutic outcomes. Standardization of dosing, delivery parameters, and rehabilitation protocols will be essential for future translational research. Preclinical models could provide initial insights into pharmacokinetics, tissue penetration, and mechanistic interactions, while well-designed clinical trials will be required to evaluate safety, efficacy, and long-term outcomes.

Accordingly, this proposed model should be interpreted as a translational and hypothesis-generating framework intended to guide future research rather than a validated therapeutic protocol. Its primary value lies in integrating pharmacological, technological, and rehabilitation-based concepts into a unified approach that may better address the multifactorial nature of peripheral neuropathy.

**Figure 2:**
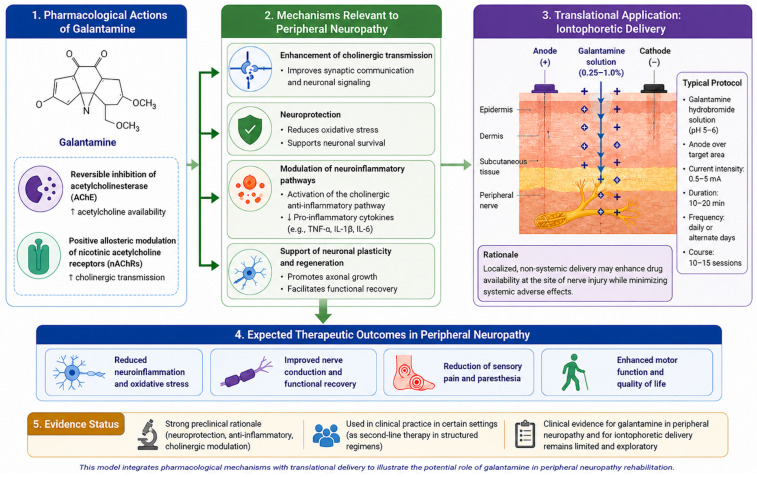
Mechanistic and translational model of galantamine in peripheral neuropathy.

The proposed multimodal interaction and its potential mechanistic pathways are illustrated in Figure 2.

## 8. Safety and Translational Considerations

Potential safety considerations should be carefully evaluated when proposing the combined use of galantamine, iontophoresis, and Black Sea brine in peripheral neuropathy. Given the multimodal nature of this approach, safety must be considered at the level of each individual component as well as their potential interactions. Key safety considerations are summarized in Table 3.

Iontophoresis is generally regarded as a safe and non-invasive technique; however, it is not without risks. Local adverse effects such as skin irritation, erythema, discomfort, and, in rare cases, superficial burns may occur, particularly when excessive current intensity, prolonged exposure, or improper electrode placement is used [13,35,36]. Electroosmotic flow and uneven current distribution may further contribute to variability in drug penetration and local tissue exposure [34]. In addition, interindividual variability in skin resistance, hydration, and barrier integrity may significantly influence both safety and delivery efficiency, highlighting the need for individualized parameter adjustment and careful monitoring [46].

In the context of galantamine, safety considerations are primarily related to uncertain pharmacokinetics and potential systemic exposure. While transdermal and iontophoretic delivery aim to localize drug action, it remains unclear to what extent galantamine may be absorbed systemically, particularly with repeated applications. This raises concerns regarding possible cholinergic adverse effects, including gastrointestinal symptoms, bradycardia, and central nervous system effects [29,45]. Furthermore, the physicochemical properties of galantamine, including pH sensitivity and potential instability in solution, may affect dosing accuracy and reproducibility [14,16]. These uncertainties underscore the need for dedicated pharmacokinetic and pharmacodynamic studies in transdermal delivery settings [15].

Black Sea brine, although widely used in rehabilitation medicine, also requires careful consideration of safety parameters. High mineral concentration and osmotic effects may lead to skin irritation or hypersensitivity reactions, particularly in patients with compromised skin integrity or dermatological conditions [39,42]. In addition, variability in mineral composition across geographic sources may influence both therapeutic effects and tolerability, emphasizing the importance of standardization and quality control [40]. Temperature and duration of exposure represent additional variables that may impact safety and should be carefully controlled in clinical protocols.

From a combined therapy perspective, the interaction between iontophoresis, pharmacological agents, and mineral-rich environments remains largely unexplored. The simultaneous application of electrical current, active pharmacological compounds, and hypertonic mineral solutions may alter skin permeability, drug transport dynamics, and local tissue responses in unpredictable ways [35]. This introduces an additional layer of uncertainty regarding both safety and efficacy, reinforcing the need for preclinical validation.

General contraindications associated with electrotherapy and topical interventions must also be considered. These include damaged or inflamed skin, presence of implanted electronic devices (e.g., pacemakers), pregnancy, and unstable cardiovascular or systemic conditions [35,48]. Appropriate patient selection and adherence to established electrotherapy safety guidelines are essential to minimize risk.

From a translational perspective, the absence of standardized protocols represents a major limitation. The development of this multimodal approach will require rigorous standardization of drug formulations, iontophoretic parameters, brine composition, and treatment duration. In addition, dose–response relationships, long-term safety, and reproducibility must be systematically evaluated in both preclinical and clinical settings [16,21].

Importantly, although the potential risks described above should not be underestimated, they are generally mild and manageable within controlled clinical environments. In routine rehabilitation practice, continuous patient monitoring, individualized adjustment of treatment parameters, and adherence to safety protocols can significantly reduce the likelihood of adverse events. Nevertheless, the lack of high-quality clinical evidence necessitates a cautious and stepwise translational approach.

## 9. Limitations of Current Evidence

Several limitations of the current evidence should be acknowledged.

First, no clinical trials have directly evaluated the combined use of galantamine, iontophoresis, and Black Sea brine in the treatment of peripheral neuropathy. As a result, the proposed multimodal approach remains untested in controlled clinical settings.

Second, the available evidence is largely derived from indirect sources, including mechanistic reasoning, preclinical studies, and investigations of individual interventions rather than their combined application. This limits the ability to draw definitive conclusions regarding clinical efficacy.

Third, part of the literature includes regional and historical sources which, although valuable for understanding clinical practice and therapeutic traditions, may not fully meet contemporary methodological standards.

Finally, the proposed model relies in part on theoretical integration of pharmacological and rehabilitation-based mechanisms and should therefore be interpreted as hypothesis-generating rather than evidence-confirmed.

Taken together, these limitations warrant cautious interpretation and emphasize the need for well-designed experimental and clinical studies.

## 10. Future Research Directions

Future research should aim to systematically evaluate the proposed multimodal approach within a staged translational framework.

Initial studies should focus on preclinical models of peripheral neuropathy, including diabetic, traumatic, and inflammatory models, to assess the combined effects of galantamine, iontophoresis, and mineral-based therapy.

Subsequently, skin permeation and pharmacokinetic studies are required to characterize the transdermal delivery profile of galantamine, including dosing accuracy, tissue distribution, and reproducibility of delivery.

Early-phase pilot clinical studies should then evaluate safety, tolerability, and feasibility in human subjects.

If preliminary findings are favorable, controlled randomized clinical trials will be necessary to assess therapeutic efficacy and to compare the proposed approach with current standard-of-care treatments.

Finally, future research should aim to identify specific neuropathy subtypes that may benefit most from this intervention, given the heterogeneity of peripheral neuropathy and its diverse pathophysiological mechanisms.

## 11. Conclusions

The combination of galantamine, iontophoresis, and Black Sea brine represents a novel and conceptually grounded multimodal approach to peripheral neuropathy management. The proposed model integrates pharmacological, physical, and rehabilitation-based mechanisms that may address complementary aspects of peripheral nerve dysfunction.

However, despite this biological plausibility, direct clinical evidence supporting the efficacy of the combined intervention is currently lacking. The available data remain indirect, heterogeneous, and largely derived from investigations of the individual components rather than their integrated application.

Therefore, this approach should be considered investigational and hypothesis-generating rather than clinically established. Further preclinical and clinical studies are required to evaluate its safety, feasibility, and therapeutic potential before evidence-based recommendations can be made.

If validated, this multimodal strategy may offer a basis for more targeted and integrated rehabilitation approaches in selected patients with peripheral neuropathy.

## Figures and Tables

**Table 1:** Clinical and experimental evidence for brine-based therapy.

Study	Condition	Intervention	Outcome	Limitations
Grozeva and Stoicheva 2015 [43]	Diabetic polyneuropathy Thalassotherapy including	Black Sea brine applications	Improvement in pain and functional status	Non-randomized design; regional study; small sample size
Koleva 2011 [33]	Neurological and musculoskeletal conditions	Brine-based rehabilitation (baths, compresses, electrophysiotherapy)	Reported improvements in circulation, pain, and function	Descriptive source; lacks controlled clinical data
Mikhaylenko et al. 2020 [11]	Spinal disorders	Sanatorium-based rehabilitation including mineral therapies	Functional improvement and symptom relief	Mixed interventions; no isolated brine effect
Maraver et al. 2025 [39]	Thalassotherapy/mineral environments	Evaluation of mineral-rich saline environments	Potential benefits on circulation and recovery	Not disease-specific; indirect evidence
Puszcz et al. 2026 [42]	Rehabilitation and lifestyle medicine	Balneotherapy and physiotherapy approaches	Anti-inflammatory and quality-of-life improvements	Not specific to neuropathy; heterogeneous population

**Table 2:** Evidence overview of iontophoresis-based interventions in neuropathic conditions.

Agent/Modality	Target Condition	Study Type	Key Findings	Limitations	Ref.
Galantamine (iontophoresis)	Peripheral neuropathy (conceptual)	Narrative/conceptual studies	Proposed as a non-systemic delivery strategy; no clinical trials available	No clinical validation; limited to theoretical and non-indexed reports	Ivanova [9]
S(+)-Ketamine (iontophoresis)	Central neuropathic pain	Placebo-controlled trial	No significant reduction in pain intensity; some improvement in quality of life	Not specific to peripheral neuropathy; small sample size	Vranken [18]
Local anesthetics (iontophoresis)	Experimental sensory models	Experimental study	Achieved reversible local anesthesia and sensory blockade	No direct clinical application in neuropathy	Rawat [13]
Iontophoresis (general use)	Carpal tunnel syndrome	Clinical observational study	Used as adjunct therapy; some symptom improvement reported	Limited methodological rigor; heterogeneous outcomes	Kurek [47]
Iontophoresis (general)	Peripheral neuropathy rehabilitation	Review/rehabilitation reports	Mentioned as supportive modality in rehabilitation protocols	Lack of controlled trials; indirect and heterogeneous evidence	Ivanova, Kurek [9,47]

**Table 3:** Safety considerations of the proposed multimodal therapy.

Component	Potential Risk	Mechanism/Cause	Clinical Considerations
Iontophoresis	Skin irritation, erythema, superficial burns	Electrical current intensity, prolonged exposure, uneven current distribution	Adjust current intensity; monitor skin condition; avoid damaged skin
Iontophoresis	Variability in drug delivery	Differences in skin resistance, hydration, and electrode placement	Standardization of protocol required; individualized dosing
Galantamine (local/systemic exposure)	Uncertain systemic absorption; cholinergic side effects	Transdermal penetration and unclear pharmacokinetics	Need for pharmacokinetic studies; cautious dose titration
Galantamine (formulation)	Instability and pH sensitivity	Chemical properties affecting ionization and delivery efficiency	Controlled formulation and buffering required
Black Sea brine	Skin irritation or hypersensitivity	High mineral concentration and osmotic effects	Adjust concentration and exposure time; avoid in compromised skin
Black Sea brine	Variability in composition	Differences between geographic sources and preparation methods	Standardization and quality control needed
Combined therapy	Unpredictable interaction effects	Lack of data on combined pharmacological and physical interventions	Requires preclinical validation and controlled clinical studies
General contraindications	Adverse events in vulnerable populations	Electrotherapy and topical exposure risks	Avoid in pacemakers, pregnancy, unstable conditions

## Data Availability

Data are contained within the article.

## Abbreviations

The following abbreviations are used in this manuscript:

NHIF	National Health Insurance Fund
PMR	Physical medicine and rehabilitation
